# The relationship between health-promoting lifestyle and sleep quality in postmenopausal women

**DOI:** 10.1051/bmdcn/2018080211

**Published:** 2018-05-28

**Authors:** Asieh Moudi, Ali Dashtgard, Hamid Salehiniya, Maryam Sadat Katebi, Mohammad Reza Razmara, Mohammad Reza Jani

**Affiliations:** 1 Department of Midwifery, Ahvaz Jundishapur University of Medical Sciences Ahvaz Iran; 2 Department of Nursing, Lecturer of Nursing and Midwifery School, Birjand University of Medical Sciences Birjand Iran; 3 Zabol University of Medical Sciences Zabol Iran; 4 Department of Epidemiology and Biostatistics, School of Public Health, Teheran University of Medical Sciences Teheran Iran; 5 Department of Midwifery, Lecturer of Nursing and Midwifery School, Birjand University of Medical Sciences Birjand Iran

**Keywords:** Lifestyle, Menopause, Sleep, Sleep disorder, HPL: Health-promoting lifestyle, HRT: Hormone, Replacement Therapy, PSQI: Pittsburgh Sleep, Quality Index, HPLP2: Health-Promoting, Lifestyle Profile II

## Abstract

Background: Menopausal women are widely reported to have poor sleep quality and sleep problems. It is not clear whether increases in sleep disturbance are brought about by hormone changes associated with menopause or due to psychosocial and physical problems.

Method: This cross-sectional study was conducted on 600 menopausal women aged between 40 and 60 without any known severe illnesses in the city of Qaen, Iran, from April 2015 to May 2016. Data were collected by Health-Promoting Lifestyle Profile II and Pittsburgh Sleep Quality Index.

Main outcome measures: This study was conducted to investigate the relationship between health-promoting lifestyle and sleep quality in Iranian postmenopausal women. Data were analyzed using an independent t-test, Mann-Whitney, *Chi*-square, Spearman and univariate logistic regression.

Result: The univariate logistic regression suggested that the physical activity dimension of lifestyle (OR = 1.095, 95% CI: 1.035-1.158, *P* < 0.006), non-smoking status (OR = 0.549, 95% CI: 0.331-0.912, *P* < 0.021) and occupation (women who were farmer compared with housewives) (OR = 0.239, 95% CI (0.074-0.775), *P* < 0.017) were associated with sleep quality.

Conclusion: Postmenopausal women in this study were at high risk for poor sleep quality. Poor sleep quality was associated with low levels of physical activity, smoking and being a housewife compared to being a farmer. Therefore, there is an essential need to educate women about health-promoting behaviors including daily physical activity and avoiding smoking which are associated with quality of sleep.

## 1. Introduction

Menopause is the end of menstrual period. Physiologically, it is due to natural discharge of ovarian follicular function, a situation interpreted with permanent amenorrhea and generally related to aging [1, 2].

Among American and European populations, menopause occurs between 48-54 years and the average is 51.4 [3, 4]. However, the average age of menopause has been reported between 46.9 and 49 years in different parts of Iran, which is lower than that in the other countries [5]. Therefore, more than one-third of women’s life is spent after menopause [6, 7].

In recent decades, due to medical advances and increased life expectancy in the world, about 95 percent of women experience menopause [8]. It is anticipated that the number of postmenopausal women will have increased from 467 million to 1,200,000,000 people across the world by 2030, and the largest increase will occur in the developing countries [9].

During menopause, the ovaries stop producing female sex hormones, most specifically estrogen [9]. Reduction of estrogen causes hot flashes, night sweats, palpitations, headache, dizziness, fatigue, irritability and sleep disturbances [10-14]. Among these, sleep disturbances are quite common among postmenopausal women [15]. About one-third of adults and 40-60 % of postmenopausal women suffer from sleep problems [12, 14, 16]. Sleep disorders are one of the main reasons for referring postmenopausal women to health centers and the use of tranquilizers [17, 18].

Some studies suggest that menopause symptoms are of individual character and in addition to hormonal and biological changes, they are caused by external factors: lifestyle, state of health, social functioning, perception of aging, culture, and race/ ethnicity [1, 19-23]. However, it is not clear whether increases in sleep disturbance are brought about by hormone changes associated with menopause or due to psychosocial and physical problems [24, 25].

Lifestyle is the typical manner and traditional daily activities that people live with, and these conditions can affect the health of individuals [26]. Health-promoting lifestyle (HPL) includes behaviors such as nutrition, physical activity, responsibility for health, stress management, interpersonal relationships and spiritual growth [12].

Physical activity is one of the lifestyle’s factors that may affect sleep quality. Some studies have suggested that postmenopausal women with physical activity had better sleep [21, 27], whereas others have not [28].

Allen (2004) showed that a high body mass index, stress and smoking are related to experiencing more sleep problems in postmenopausal women [12]. However, Chung suggested that sleep disorders in menopausal women were not associated with their lifestyle [24].

Due to the contradictory results of the aforementioned studies and the fact that few studies have been conducted exclusively on the relationship between health-promoting lifestyle and sleep quality in postmenopausal women, this study was conducted to investigate the relationship between health-promoting lifestyle and sleep quality in Iranian postmenopausal women. Identifying HPL factors affecting better sleep among postmenopausal women may enable physicians to prevent the development of sleep disturbances at menopause.

## 2. Methods

This cross-sectional study was conducted on 600 menopausal women referred to the gynecology clinic of Shohada Hospital of Birjand University of Medical Sciences in Qaen, Iran, from April 2015 to May 2016.

Postmenopausal women who had sleep problems were recruited by convenient sampling from among postmenopausal women referring to the gynecologic clinic of the hospital. Inclusion criteria were postmenopausal women aged between 40-60 whose last menstrual period was at least 1-5 years, and normal menopause. Exclusion criteria included women who were under medication for anti-depressants, hypnotics, phytoestrogens and hormone replacement therapy (HRT), and those with a mental illness, abnormal mass in the breast and/or any abnormality in the thyroid.

This study was approved by the Ethics Committee of Birjand University of Medical Sciences, (44/94). Women were free to participate or not in the study and were enrolled after obtaining written informed consent, and all the information was collected and kept confidential.

### 2.1 Sample size calculation

The sample size was calculated by comparing the mean formula of the lifestyle score at the pilot study. It was calculated to be 472 by 95% confidence interval and power of 80%, and 20% loss. Then the final sample size was determined to be 600.

N=(Z1−α2+Z1−β)2(S12+S22)(μ1−μ2)=(1.96+0.84)2⌊(4.75)2+(5.32)2⌋(21.6−20.3)=236

### 2.2 Measurements

Study tools included self-structured questionnaires for sociodemographic data, reproductive history and factors affecting sleep quality, hot flashes and night sweats checklist, Pittsburgh Sleep Quality Index (PSQI) and Health-Promoting Lifestyle Profile II (HPLP2).

The questionnaires of the socio-demographic, reproductive history and factors affecting sleep were completed at the beginning of the study.

Hot flash daily checklist was used to record hot flashes (including intensity, duration and frequency). Night sweats daily record tool was completed to record the frequency and severity of night sweats (mild, moderate and severe) every morning after waking up.

The validity of socio-demographic questionnaire and checklists were assessed by content validity. Hot flash and night sweat checklists were completed for 2 weeks during study.

PSQI is a self-report scale consisting of 7 components and 19 questions. The components include subjective sleep quality (C1), sleep latency (C2), sleep duration (C3), habitual sleep efficiency (C4), sleep disturbances (C5), use of sleeping medications (C6) and daytime dysfunction (C7). The score ranges of this questionnaire is 0-21 and an overall score of more than 5 indicates poor quality of sleep [9]. PSQI studies quality of sleep in recent month. The validity and reliability of this questionnaire was assessed and approved by Moghadam et *al*. in Iran [29].

HPLP II includes 52 items the scoring of which was done by the 4-item Likert scale where *never* scored 1, *sometimes* scored 2, *often* scored 3, and *always* scored 4. The HPLPII has six subscales of health responsibility (9 items), physical activity (8 items), nutrition (9 items), spiritual growth (9 items), interpersonal relations (9 items) and stress management (8 items). Total score ranges from 52 to 208 [12]. The validity and reliability of this questionnaire was assessed by Mohammadi Zeidi in Iran [30].

### 2.3 Statistical analysis

Data were analyzed using SPSS statistical software version 24. To describe the characteristics of the subjects, descriptive statistics, indicators of central tendency and dispersion (mean and standard deviation), and frequency were used. Objectives of the study were investigated using an independent t-test, Mann-Whitney, *Chi*-square, Spearman and univariate logistic regression. Results from the logistic regressions were presented as odds ratios (ORs) with 95% confidence intervals (95% CI).

The goodness of fit of the model was tested with the Hosmer and Lemeshow procedure.

## 3 Results

The mean age of the participants was 54.4 ± 4.75 years and mean age of menopause was 47 ± 3.91 years. Most of the postmenopausal women were illiterate (65%), married (76.5%), and housewife (86%). Eighty-three percent of them did not use tobacco and 84 percent did not do exercise at all. Socio-demographic characteristics of the participants are shown in Table 1.

**Table 1:** Socio-demographic characteristic of participants.

Characteristics	Sleep quality Mean ± SD or N (%)	
	Good (≤5) N = 262	Poor (>5) N = 338	*P* value
Age (y)	54.0 ± 4.88	54.7 ± 4.63	0.055
Age of menopause onest (y)	46.86 ± 3.86	47.25 ± 3.94	0.219
Gravida	6.9 ± 3.29	6.9 ± 3.22	0.957
Parity	5.9 ± 2.80	6.1 ± 2.75	0.549
BMI	25.0 ± 4.89	26.0 ± 5.23	0.017
Duration of smoking (y)	1.5 ± 5.27	2.8 ± 6.64	0.012
Frequency of sweating	1.7 ± 0.97	1.9 ± 1.24	0.029
**Hot flashes**			
Asymptomatic	70 (45.8)	83 (54.2)	0.189
Mild	90 (46.2)	105 (53.8)	
Moderate	62 (42.2)	85 (57.8)	
Sever	40 (38.1)	65 (61.9)	
**Sweating**			
Mild	164 (46.2)	191 (53.8)	0.051
Moderate	73 (44.5)	91 (55.5)	
Sever	25 (30.9)	56 (69.1)	
**Education**			
Illiterate	163 (41.7)	228 (58.3)	0.298
Primary school	88 (48.1)	95 (51.9)	
Secondary school	4 (33.3)	8 (66.7)	
Diploma	7 (50.0)	7 (50.0)	
Academic	163 (41.7)	228 (58.3)	
**Marital status**			
Single	203 (44.2)	256 (55.8)	0.557
Married	1 (16.7)	5 (83.3)	
Widowed	54 (43.5)	70 (56.5)	
Divorced	4 (36.4)	7 (63.6)	
**Occupation**			
Farmer	229 (43.7)	295 (56.3)	0.092
Employee	4 (18.2)	18 (81.8)	
Worker	16 (51.6)	15 (48.4)	
Housewife	13 (56.5)	10 (43.5)	
**Smoking**			
Yes	30 (29.4)	72 (70.6)	0.001
No	232 (46.6)	266 (53.4)	
**Exercise**			
Yes	47 (50.5)	46 (49.5)	0.146
No	215 (42.4)	292 (57.6)	

The mean global PSQI Score of the women was 6.7 ± 3.41. More than half of the postmenopausal women (56.3%) had poor sleep quality and 348 (58%) had daytime sleepiness. Poor sleep quality and daytime sleepiness were not significantly correlated (Spearman rank, r = -0.007, *P* = 0.863).

The outcomes of HPLP II measurement showed relatively low scores for both physical activity (13.7 ± 4.52) and stress management (18.1 ± 3.22). The total mean of HPLP II score was 122.8±17.80. The total score of lifestyle was significantly higher in women with good sleep quality as opposed to women with poor sleep quality (125.9 ± 16.75 vs. 120.4 ± 18.23, *p* < 0.001) (Table 2). The score for all dimensions of HPLP II except interpersonal relations was significantly higher in women with good sleep quality (Table 2). In addition, sleep quality was significantly associated with body mass index, smoking, duration of smoking and the frequency of sweating (Table 1).

**Table 2:** The mean scores of lifestyles, in menopausal women with good and poor sleep quality.

Variable	Quality of sleep Mean ± SD		*P value*
	Good (≤5) N = 262	Poor (>5) N = 338	
Interpersonal relations	53.80 ± 23.8	3.97 ± 23.8	0.850
Nutrition	4.19 ± 24.3	4.29 ± 23.1	0.001
Health responsibility	4.85 ±21.3	5.39 ±20.4	0.034
Physical activity	4.31± 14.7	4.51 ± 12.9	0.001
Stress management	3.01 ± 18.5	3.33 ± 17.8	0.007
Spiritual growth	4.07 ± 23.2	4.53 ± 22.5	0.042
Total score of lifestyle	16.75 ± 125.9	18.23 ± 120.4	0.001

Table 3 shows univariate relationships between independent variables and poor sleep quality. The univariate logistic regression suggested that physical activity was strongly associated with quality of sleep in women with poor sleep quality who were more likely to have a lower score of physical activity (OR = 1.095, 95% CI (1.035-1.158), *P* < 0.006). Non-smokers (OR = 0.549, 95% CI (0.331-0.912), *P* < 0.021) were less likely to be poor sleepers. Women who were farmers compared with housewives (OR = 0.239, 95% CI (0.074-0.775), *P* < 0.017) had a lower risk for poor sleep quality.

**Table 3:** Factors associated with poor sleep quality in postmenopausal women.

Variable	Quality of sleep Mean ± SD or N(%)		Odds ratio (95% CI)	*P value*
	Good (≤5)	Poor (>5)		
Age	4.88 ± 54.0	4.63 ± 54.7	0.967 (0.927-1.009)	0.121
Age of menarche	1.58 ± 13.4	1.57 ± 13.3	1.057 (0.941-1.186)	0.350
Age onset of menopause	3.86 ± 46.9	3.94 ± 47.3	0.984 (0.937-1.033)	0.510
**Smoking**				
Yes*	30 (29.4)	72 (70.6)		
No	232 (46.6)	266 (53.4)	0.549 (0.331-0.912)	0.021
Frequency of hot flashes			1.065 (0.980-1.158)	0.137
Frequency of sweating			0.882 (0.725-1.072)	0.207
Interpersonal relations dimension	3.80 ± 23.8	3.97 ± 23.8	0.993 (0.937-1.052)	0.812
Nutrition dimension	4.19 ± 24.3	4.29 ± 23.1	1.048 (0.997-1.101)	0.064
Health responsibility dimension	4.85 ± 21.3	5.39 ± 20.4	0.977 (0.936-1.021)	0.301
Physical activity dimension	4.31 ± 14.7	4.51 ± 12.9	1.095 (1.035-1.158)	0.001
Stress management dimension	3.01 ± 18.5	3.33 ± 17.8	0.995 (0.923-1.072)	0.888
Spiritual growth dimension	4.07 ± 23.2	4.53 ± 22.5	0.996 (0.943-1.053)	0.897
BMI	25.0 ± 4.94	26.0 ± 5.23	0.974 (0.940-1.010)	0.152
**Daytime sleepiness**				
Yes*	153 (44.0)	195 (56.0)		
No	109 (43.3)	143 (56.7)	1.141 (0.790-1.648)	0.481
Marital status				
Married*	203 (44.2)	256 (55.8)		
Single	1 (16.7)	5 (83.3)	0.344 (0.036-3.319)	0.356
Widowed	54 (43.5)	70 (56.5)	1.016 (0.645-1.599)	0.946
Divorced	4 (36.4)	7 (63.6)	1.022 (0.273-3.822)	0.975
**Hot flashes**				
Asymptomatic*	70 (45.8)	83 (54.2)		
Mild	90 (46.2)	105 (53.8)	0.722 (0.435-1.198)	0.207
Moderate	62 (42.2)	85 (57.8)	0.658 (0.371-1.170)	0.154
Sever	40 (38.1)	65 (61.9)	0.602 (0.305-1.187)	0.143
**Sweating**				
Mild*	164 (46.2)	191 (53.8)		
Moderate	73 (44.5)	91 (55.5)	0.907 (.584-1.410)	0.665
Sever	25 (30.9)	56 (69.1)	0.670 (0.348-1.289)	0.230
**Education**				
Illiterate*	163 (41.7)	228 (58.3)		
Some high school or less	88 (48.1)	95 (51.9)	1.121 (0.746-1.685)	0.583
Graduated from high school	8 (66.7)	4 (33.3)		0.444
Graduated from college	7 (50.0)	7 (50.0)		0.732
**Occupation**				
Housewife*	295 (56.3)	229 (43.7)		
Farmer	18 (81.8)	4 (18.2)		0.017
Worker	15 (48.4)	16 (51.6)		0.540
Employee	10 (43.5)	13 (56.5)		0.583

* Reference category.

## 4 Discussion

The present study was designed to determine the relationship between health-promoting lifestyle and sleep quality in Iranian postmenopausal women. Results of this study showed that, poor sleep quality was common in women and it was related to sociodemographic factors and lifestyle. After adjusting for other confounders, poor sleep quality was associated with low levels of physical activity, smoking and being a housewife compared to being a farmer. Low physical activity was the strongest independent risk factor for poor sleep quality in this sample of women.

In this research, more than half of postmenopausal women had poor sleep quality. This finding was in concordance with the study by Azhari [31] in Iran and a study by Hung in Taiwan [24]. In comparison with American women [23] and Latin American women [21], the samples of this study had poor sleep quality. The different results may be due to biological, socio-economical, psychosocial, cultural, and race/ethnic factors.

Our participants had a moderate health-promoting lifestyle which corroborates other studies conducted in Iran [31-34] and China [35]. However, they were better than Korean middle-aged women [36] and Turkish worker women [37], which is probably the result of cultural differences in health behaviors between people from different countries.

However, the methodological difference in these studies to assess sleep quality and lifestyle has made it difficult to compare them; thus, these studies need to be carefully interpreted.

The relationship between physical activity and sleep quality is consistent with that of a great deal of previous studies [20, 33, 38, 39], although in some studies there is no evidence to support this [31, 35, 40]. It seems the difference between the age ranges of the study population and the tools employed are the possible causes of this disagreement. The age range in our study was 40-60 years but it was 20-40 years in Cheng’s study. In addition, insomnia in Cheng’s study was assessed by one “Yes” or “No” questions: e.g., do you often suffer from insomnia [35].

In the present study interpersonal relationship was not associated with sleep quality. This result is in agreement with that of Azhari (2014) and Cheng (2015) although it differs from Shin’s study (2007) [31, 35, 36]. The meaning of interpersonal relations in women of Shin’s study conflicts with a mother-in-law. This is because, in Korea, the majority of elders (60%) live with their children, negative relationship with a mother-in-law affects other relationships with family members and worsens mental health [36].

Lack of any relationship between nutrition and sleep quality in the present study agreed with the results of Cheng (2015) [35], but it was against Shin (2007) and Azhari (2013) [31, 36]. The disagreement between studies is probably due to different independent variables being considered in the univariate models. In our study, dimensions of lifestyle along with other variables were included in the univariate model for sleep quality but were not included in the study by Azhari [31].

Health responsibility was not associated with sleep quality. This finding corroborates the results of Azhari (2013) and Cheng (2015), but is in contrast to Shin’s finding (2007) [31, 35, 36]. These differences may be due to the fact that the women participating in the present study had lower health responsibility than those in Shin (20.8 ± 5.20 vs. 35.7 ± 7.83).

There was no association between stress management and sleep quality. This also accords with observations of Cheng (2015) and Shin (2007); however, it differs from Azhari’s findings [31, 35, 36]. The discrepant results might be attributable to the different genetic vulnerability for insomnia in the participants [1].

No association was observed between spiritual growth and quality of sleep in the present study. This result confirms the observations of Azhari and Shin but is against the findings of Cheng [31, 35, 36]. Spiritual growth represents the latent abilities of individuals to complete themselves in order “to be the best that they are efficient at doing”. Expression of one’s innovation, the quest for spiritual insight, the pursuit of knowledge, and the desire to give to the society are examples of self-actualization [35]. Thus, contradictory results might be attributable to different age and education of the participants because participants of our study were 40-60 years and mostly illiterate while those of Cheng’s study were 20-40 years and mostly academic.

The women who smoke more often have poor sleep quality. Although this finding supports previous research into this area [8, 22, 41, 42], it differs from some published studies [20, 24, 43]. The association between heavy smoking and poor sleep quality has been explained in some studies [20]. Hence, Iranian women smoke more tobacco which is equivalent to being heavy smokers, and this seems to be the reason for the observed difference.

Women who were housewives compared with farmers had poor sleep quality. This result is probably due to the positive impact of physical activity on sleep quality [21] because farmer women do more physical activity than housewives.

## 5 Limitations

Our study limitations were included: firstly, due to the crosssectional design of this study, it is not possible to assess the causal relationship between lifestyle and sleep quality. Secondly, because all information was collected by self-reported questionnaires, there is the possibility of information bias. Consequently, the findings should be cautiously generalized to other populations of women. Thirdly, this study did not compare the relationship between sleep quality and lifestyle in postmenopausal and menstrual women. A longitudinal study design or the comparison between menstrual and menopausal women is recommended in future studies.

## 6 Conclusion

Postmenopausal women in this study were at high risk for poor sleep quality. Physical activity dimension of HPLP-II, smoking and being a housewife compared to being a farmer were associated with poor sleep quality. There is an essential need to educate women about health-promoting behaviors, including daily physical activity and smoking cessation, which are associated with quality of sleep.

### Conflicts of interest statement

Authors declare no conflict of interest.
